# Effects of intravenous infusion of hydrogen-rich fluid combined with intra-cisternal infusion of magnesium sulfate in severe aneurysmal subarachnoid hemorrhage: study protocol for a randomized controlled trial

**DOI:** 10.1186/s12883-014-0176-1

**Published:** 2014-09-09

**Authors:** Satoru Takeuchi, Kentaro Mori, Hirohiko Arimoto, Kazuya Fujii, Kimihiro Nagatani, Satoshi Tomura, Naoki Otani, Hideo Osada, Kojiro Wada

**Affiliations:** Department of Neurosurgery, National Defense Medical College, 3-2 Namiki, Tokorozawa, Saitama 359-8513 Japan; Department of Neurosurgery, Japan Defense Forces Central Hospital, Tokyo, Japan; Department of Neurosurgery, Mishuku Hospital, Tokyo, Japan

**Keywords:** Subarachnoid hemorrhage, Early brain injury, Vasospasm, Delayed cerebral ischemia, Hydrogen-rich fluid, Oxidative stress, Magnesium, Cerebrospinal fluid

## Abstract

**Background:**

The failures of recent studies intended to prevent cerebral vasospasm have moved the focus of research into delayed cerebral ischemia away from cerebral artery constriction towards other mechanisms. Recent accumulating evidence has suggested that early brain injury is also involved in the development of delayed cerebral ischemia, and that hydrogen can prevent early brain injury. Therefore, we have established a combination therapy of intravenous hydrogen infusion and intra-cisternal magnesium sulfate infusion for the treatment of both early brain injury and cerebral vasospasm. The present randomized controlled clinical trial is designed to investigate the effects of this novel therapeutic strategy on the occurrence of cerebral vasospasm, delayed cerebral ischemia, and clinical outcomes after high-grade subarachnoid hemorrhage.

**Methods:**

This study is a randomized, double-blind, placebo-controlled design to be conducted in two hospitals. A total of 450 patients with high-grade subarachnoid hemorrhage will be randomized to one of three arms: (i) Mg + H_2_ group, (ii) Mg group, and (iii) control group. Patients who are assigned to the Mg + H_2_ group will receive intra-cisternal magnesium sulfate infusion (2.5 mmol/L) at 20 mL/h for 14 days and intravenous hydrogen-rich fluid infusion (200 mL) twice a day for 14 days. Patients who are assigned to the Mg group will receive intra-cisternal magnesium sulfate infusion (2.5 mmol/L) at 20 mL/h for 14 days and intravenous normal glucose-electrolyte solution (200 mL) without added hydrogen twice a day for 14 days. Patients who are assigned to the control group will receive intra-cisternal Ringer solution without magnesium sulfate at 20 mL/h for 14 days and intravenous normal glucose-electrolyte solution (200 mL) without added hydrogen twice a day for 14 days. Primary outcome measures will be occurrence of delayed cerebral ischemia and cerebral vasospasm. Secondary outcome measures will be modified Rankin scale score at 3, 6, and 12 months and biochemical markers.

**Discussion:**

The present protocol for a randomized, placebo-controlled study of intravenous hydrogen therapy with intra-cisternal magnesium infusion is expected to establish the efficacy and safety of this therapeutic strategy.

**Trial registration:**

UMIN-CTR: UMIN000014696

## Background

Aneurysmal subarachnoid hemorrhage (SAH) accounts for 5% of all strokes [1]. The mortality rate is approximately 50%, and 20% to 30% of surviving patients have significant neurologic deficits [2,3]. In particular, high-grade SAH, which is classified as Hunt and Kosnik grades 4 and 5, accounts for approximately 20-40% of patients with SAH, and its prognosis is extraordinarily poor [4]. Delayed cerebral ischemia (DCI), a clinical diagnosis previously proposed by Vergouwen et al., is considered to be the most important cause of mortality and morbidity [5]. Angiographic cerebral vasospasm develops in approximately 70% of patients between 4 and 14 days after SAH, and the primary mechanism underlying DCI was widely believed to be cerebral vasospasm [1,6].

Numerous experimental and clinical studies have been conducted to prevent and/or treat cerebral vasospasm, and various prophylactic strategies against cerebral vasospasm have been advocated [7–12]. For example, magnesium sulfate has been studied as one of the most attractive therapeutic agents for decades [10–18]. Magnesium sulfate exhibits several beneficial effects such as vasodilation of vessels and attenuation of neuronal death. The mechanisms of these effects include blockage of the voltage-dependent calcium channels, inhibition of excitatory glutamate release, and interference with N-methyl-D-aspartate-glutamate receptors [19]. Several randomized controlled trials (RCTs) have been conducted to investigate the effects of intravenous magnesium sulfate administration on outcome after SAH [12–18]. However, these RCTs and meta-analyses showed that magnesium sulfate did not decrease DCI or improve the poor functional outcome after SAH [10–18].

Two main reasons for these negative results have been identified. Firstly, intravenous magnesium sulfate infusion results in only limited increases in cerebrospinal fluid (CSF) magnesium levels, whereas serious adverse events (such as bradycardia and hypotension) can occur at serum magnesium levels >2 mmol/L [20]. Therefore, these contradictory effects may be difficult to overcome for clinical use of intravenous magnesium infusion. Secondly, recent accumulating findings have suggested that early brain injury (EBI) is involved mainly in the development of DCI and causes the high mortality and morbidity observed after SAH [21,22]. EBI is the product of pathological mechanisms triggered in the brain during the first 72 hours after SAH. However, the time from the onset to initiation of intravenous magnesium administration was approximately 30–40 hours in most RCTs, whereas more time is required to achieve significant CSF magnesium levels after infusion [12,18,23]. This time latency can also be considered as one of the reasons for the negative results in RCTs.

Previously, we studied the safety and efficacy of intra-cisternal magnesium sulfate infusion and found that significant increases in CSF magnesium levels can be achieved without changes in serum levels [23–25]. However, the time latency from infusion to achievement of significant CSF magnesium level was also present with intra-cisternal infusion, and remains unresolved [23]. Theoretically, magnesium can also exert effects on EBI [21,22], but we consider that effective CSF magnesium levels are difficult to achieve during the period of EBI development, regardless of administration route. Therefore, other treatment strategies against EBI are more promising.

Increasing evidence has suggested that enhanced oxidative stress is involved in EBI as well as cerebral vasospasm following SAH [26–29]. Hydrogen can selectively reduce hydroxyl radicals and peroxynitrites, which are very strong reactive oxygen species that react indiscriminately with nucleic acids, lipids, and proteins, resulting in DNA fragmentation, lipid peroxidation, and protein inactivation [30–38]. Previous studies have shown that hydrogen has antioxidant, anti-apoptotic, anti-inflammatory, and cytoprotective properties that are beneficial to the cell [30]. Hydrogen is highly diffusible and could potentially reach subcellular compartments, such as the mitochondria and nuclei, which are the primary sites of reactive oxygen species generation and DNA damage [30,38]. In addition, hydrogen has no side effects, and we previously showed that hydrogen can be safely administered intravenously in patients with ischemic stroke [33]. Furthermore, recent experimental studies showed that hydrogen can alleviate EBI after SAH via attenuation of neuronal apoptosis [39–42].

These findings suggest that combination therapy using intravenous hydrogen infusion and intra-cisternal magnesium sulfate infusion may be effective to treat both EBI and delayed cerebral vasospasm. The present randomized controlled clinical trial is intended to investigate the effects of intravenous hydrogen therapy with intra-cisternal magnesium sulfate infusion on the occurrences of cerebral vasospasm and DCI, and clinical outcomes in patients with high-grade SAH.

## Methods

### Overview

**Figure 1:**
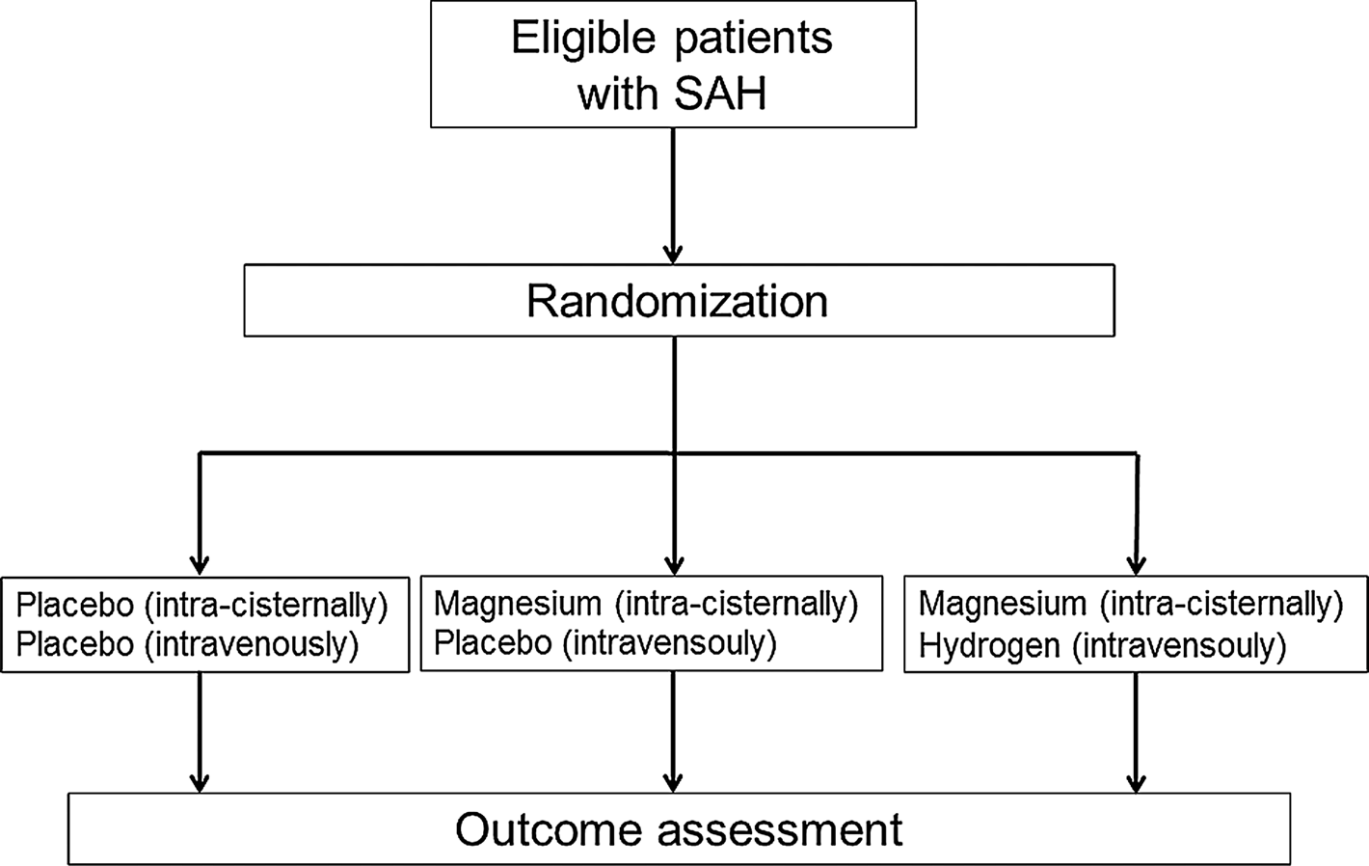
**Flow diagram.**

This study is a randomized, double-blind, placebo-controlled trial to be conducted in two hospitals (National Defense Medical College Hospital and Mishuku Hospital) and includes three arms: (i) intravenous hydrogen-rich fluid infusion with intra-cisternal magnesium sulfate infusion (Mg + H_2_ group), (ii) intra-cisternal magnesium sulfate infusion only (Mg group), and (iii) placebo (control group). The protocol was approved by the ethics committee of the National Defense Medical College in May 2013 (#1126) and was registered on UMIN-CTR (UMIN000014696). The study follows the Declaration of Helsinki and good clinical practice guidelines. Written informed consent will be obtained from each patient or family members before inclusion in the study. The procedures performed during the study are outlined in Figure 1.

### Participants

All patients presenting with a diagnosis of SAH will be checked for eligibility by the treating physician. Inclusion criteria are: 20 to 80 years of age, aneurysm rupture, Hunt and Kosnik grade 4 or 5, aneurysm treated by surgical clipping within 72 hours after the onset, and written informed consent from the patient or family member. Exclusion criteria are: severe brain edema, heart dysfunction (New York Heart Association Class III or IV), renal insufficiency (calculated creatinine clearance rate of less than 30 mL/min), Fisher grade 4 with massive intracerebral hematoma, and rejection of randomization.

### Randomization

Patients who fulfill the eligibility criteria will be assigned to either the control group, Mg group, or Mg + H_2_ group by stratified block randomization, which will be carried out using a computer system by a statistician not related to the project team to protect the double-blind design and integrity of the study. Randomization will be stratified by sex, age (20–60 years and older than 60 years), Hunt and Kosnik grade, and Fisher group. The allocation ratio will be 1:1:1. Patients, treating physicians, and investigators assessing outcomes and analyzing data will be unaware of the allocation.

### Interventions

All participating patients will be treated according to state-of-the art SAH management, comparable with recent published international guidelines [43]. During aneurysm clipping, a ventricular drainage tube will be placed in the lateral ventricle, and a cisternal drainage tube placed in the basal cistern. A spinal drainage tube will be placed in the lumber spine immediately after the clipping procedure. Fasudil hydrochloride (90 mg/day, 14 days) will be administered but not triple-H therapy. If severe cerebral vasospasm is detected by cerebral angiography at 7–10 days after surgery, fasudil hydrochloride (15 to 60 mg) will be administered via the proximal internal carotid artery [44,45].

**Figure 2:**
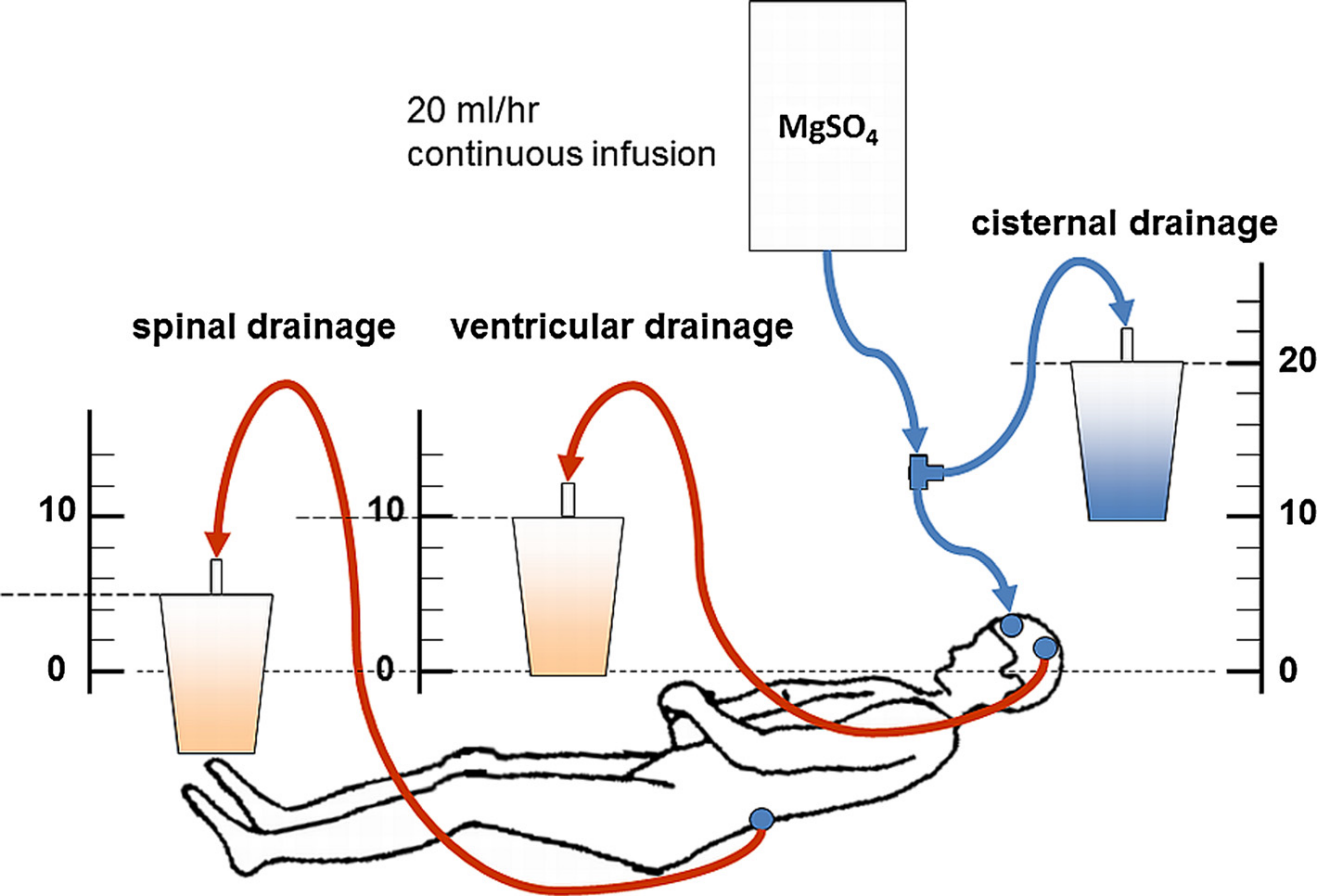
**Irrigation system.**

Patients who are assigned to the Mg + H_2_ group or the Mg group will receive intra-cisternal magnesium sulfate infusion [23]. Figure 2 illustrates the irrigation system. Continuous infusion of 2.5 mmol/L magnesium sulfate in Ringer solution will be administered at 20 mL/h for 14 days. Irrigation will be performed through the cisternal to spinal drainage. The cisternal drainage tube and the pressure control system at 20 cm H_2_O will be connected by a T-connector for safe irrigation. The ventricular drainage and spinal drainage will be set at a pressure of 10 cm H_2_O and 5 cm H_2_O, respectively, which can be adjusted by the drainage volume. The respective drainage volumes will be checked hourly to avoid overdrainage or elevated intracranial pressure. CSF and serum magnesium ion concentrations will be measured daily until 14 days after surgery, but the principal investigators of the trial will remain unaware of the results. Patients assigned to the control group will receive continuous infusion of only Ringer solution at 20 mL/h for 14 days.

Patients who are assigned to the Mg + H_2_ group will receive intravenous hydrogen-rich fluid infusion. Hydrogen-rich fluid is produced using a non-destructive hydrogen diffusion apparatus (Miz Co., Fujisawa, Japan; Patent No. 4486157, Patent Gazette of Japan 2010) as reported before [23,33]. Bags of glucose-electrolyte solution (Soldem 1, 200 mL/bag, Terumo, Tokyo, Japan) are immersed, without opening or altering the bag, in a water tank in which water is electrolyzed periodically to produce water with hydrogen concentrations of up to 1.6 ppm. The concentration of hydrogen in the bag reaches saturation, increasing to more than 1.0 ppm, because of diffusion through the wall of the bag. Additional information describing this process can be found at: http://www.e-miz.co.jp/english/technology.html#non_destructivewebsite. Intravenous hydrogen-rich fluid (200 mL) will be administered at 200 mL/h twice a day (every 12 h). Patients assigned to the control group and the Mg group will receive glucose-electrolyte solution (Soldem 1, 200 mL) without added hydrogen at 200 mL/h twice a day (every 12 h).

If severe brain edema, heart failure, or renal failure occurs, the study intervention will be stopped immediately.

### CT, CT angiography, and CT perfusion

Head computed tomography (CT) will be performed on admission, and at 3, 7, 10, and 14 days after surgery. Head CT angiography will be performed on admission and at 7 days after surgery. Head CT perfusion will be performed at 7 days after surgery. All images will be reviewed by an experienced neuroradiologist to identify the occurrence of DCI and cerebral vasospasm.

### Digital subtraction angiography

Cerebral digital subtraction angiography will be performed on admission and at 7–10 days after surgery. Digital subtraction angiography will be reviewed by an experienced neuroradiologist to identify the occurrence of cerebral vasospasm.

### Transcranial Doppler

Transcranial Doppler imaging will be performed daily until 14 days after surgery. The mean velocity of the proximal middle cerebral artery (M1) will be recorded in both hemispheres.

### Biochemical markers

Venous blood and CSF samples will be taken for assays of several biochemical markers at 1, 3, 7, and 14 days after surgery. Malondialdehyde will be assessed as an indicator of oxidative stress [33,46]. Neuron-specific enolase and S-100 calcium binding protein B will be assessed as markers of neuronal and glial injuries [21,47]. C-reactive protein will be assessed as an inflammatory marker [21,48].

### Outcome measures

Primary outcome measures will be as follows.

- Occurrence of DCI: new focal neurological deficits (motor or speech deficits) that developed after SAH, decrease in Glasgow Coma Scale of ≥2 points for >6 hours, or new cerebral infarction not related to surgery, rebleeding, progressive hydrocephalus, electrolyte or metabolic disturbance, or infection [5,15].
- Occurrence of cerebral vasospasm: angiographic vasospasm which is defined as moderate-to-severe arterial narrowing on CT angiography and/or digital subtraction angiography not attributable to atherosclerosis, catheter-induced spasm, or vessel hypoplasia. Transcranial Doppler vasospasm which is defined as a mean flow velocity in the M1 of >120 cm/s [49].

Secondary outcome measures will be as follows.

- Modified Rankin scale score at 3, 6, and 12 months.
- Biochemical markers (malondialdehyde, neuron-specific enolase, S-100 calcium binding protein B, and C-reactive protein).

### Sample size

Assuming that the incidence of DCI is 35% in the control group, 15% in the Mg group, and 5% in the Mg + H_2_ group, a total of 413 patients will be required (80% power and 2-sided α = 0.05). However, assuming approximately 10% loss to follow up, 450 patients will need to be recruited. Therefore, 150 patients must be randomized to each intervention arm.

### Statistical analysis

The study design accords to the ‘intention to treat’ principle. A test for overall comparison (e.g. analysis of variance, or if the conditions for analysis of variance are not met, a non-parametric equivalence such as the Kruskal-Wallis test) will be employed for each outcome across all three interventions, and if found to be significant, pair-wise comparisons will be made. We appreciate that many pair-wise comparisons suffer from Type I (false-positive) error, so we will adjust for multiplicity of comparisons by using steps such as Bonferroni and Tukey's procedure. The statistical procedures for pair-wise comparisons will depend on the nature of the data: for example, for dichotomous outcomes, we will use Fisher’s exact test or chi-square test as appropriate, and for continuous outcomes we will use the t-test if the observations in each arm are normally distributed; or the Mann–Whitney U-test if non-normally distributed. A value of p < 0.05 will be considered significant.

## Discussion

The present study is intended to investigate therapeutic strategy against both EBI and cerebral vasospasm to prevent the development of DCI using combination therapy with intravenous hydrogen infusion and intra-cisternal magnesium sulfate infusion. Previously we used intra-cisternal infusion of 15 mmol/L magnesium sulfate, but 20% of patients experienced respiratory suppression [23]. Therefore, we selected 2.5 mmol/L for the concentration of magnesium sulfate solution in the present study, because our recent experimental study showed that intra-cisternal infusion of 2.5 mmol/L (5 mEq/L) magnesium sulfate could prevent cerebral vasospasm after SAH [50]. We expect the intra-cisternal infusion of 2.5 mmol/L magnesium sulfate to maintain the beneficial effects and minimize side effects. In addition, the dose regimen of hydrogen-rich fluid in the present trial was based on our previous study, in which 38 patients with acute ischemic stroke received intravenous infusion of hydrogen-rich fluid, and adverse events included diarrhea in one patient (2.6%) and heart failure in one (2.6%) [33]. Heart failure was considered to be due to volume overload, so that heart dysfunction and renal insufficiency will be excluded in the present study.

The present protocol for a randomized, placebo-controlled study of intravenous hydrogen therapy with intra-cisternal magnesium infusion is expected to establish the efficacy and safety of this therapeutic strategy.

## Trial status

The study is currently ongoing.

## Abbreviations

CSF: Cerebrospinal fluid
CT: Computed tomography
DCI: Delayed cerebral ischemia
EBI: Early brain injury
RCT: Randomized controlled trial
SAH: Subarachnoid hemorrhage

## References

[1] Bederson JB, Connolly ES, Batjer HH, Dacey RG, Dion JE, Diringer MN, Duldner JE, Harbaugh RE, Patel AB, Rosenwasser RH, American Heart Association (2009). Guidelines for the management of aneurysmal subarachnoid hemorrhage: a statement for healthcare professionals from a special writing group of the Stroke Council, American Heart Association. Stroke.

[2] van Gijn J, Kerr RS, Rinkel GJ (2007). Subarachnoid haemorrhage. Lancet.

[3] Hop JW, Rinkel GJ, Algra A, van Gijn J (1997). Case-fatality rates and functional outcome after subarachnoid hemorrhage: a systematic review. Stroke.

[4] Shirao S, Yoneda H, Kunitsugu I, Ishihara H, Koizumi H, Suehiro E, Nomura S, Kato S, Fujisawa H, Suzuki M (2010). Preoperative prediction of outcome in 283 poor-grade patients with subarachnoid hemorrhage: a project of the Chugoku-Shikoku Division of the Japan Neurosurgical Society. Cerebrovasc Dis.

[5] Vergouwen MD, Vermeulen M, van Gijn J, Rinkel GJ, Wijdicks EF, Muizelaar JP, Mendelow AD, Juvela S, Yonas H, Terbrugge KG, Macdonald RL, Diringer MN, Broderick JP, Dreier JP, Roos YB (2010). Definition of delayed cerebral ischemia after aneurysmal subarachnoid hemorrhage as an outcome event in clinical trials and observational studies: proposal of a multidisciplinary research group. Stroke.

[6] Kassell NF, Sasaki T, Colohan AR, Nazar G (1985). Cerebral vasospasm following aneurysmal subarachnoid hemorrhage. Stroke.

[7] Velat GJ, Kimball MM, Mocco JD, Hoh BL (2011). Vasospasm after aneurysmal subarachnoid hemorrhage: review of randomized controlled trials and meta-analyses in the literature. World Neurosurg.

[8] Weyer GW, Nolan CP, Macdonald RL (2006). Evidence-based cerebral vasospasm management. Neurosurg Focus.

[9] Vergouwen MD, de Haan RJ, Vermeulen M, Roos YB (2010). Effect of statin treatment on vasospasm, delayed cerebral ischemia, and functional outcome in patients with aneurysmal subarachnoid hemorrhage: a systematic review and meta-analysis update. Stroke.

[10] Golan E, Vasquez DN, Ferguson ND, Adhikari NK, Scales DC (2013). Prophylactic magnesium for improving neurologic outcome after aneurysmal subarachnoid hemorrhage: systematic review and meta-analysis. J Crit Care.

[11] Wong GK, Boet R, Poon WS, Chan MT, Gin T, Ng SC, Zee BC (2011). Intravenous magnesium sulphate for aneurysmal subarachnoid hemorrhage: an updated systemic review and meta-analysis. Crit Care.

[12] Mees SM, Algra A, Vandertop WP, van Kooten F, Kuijsten HA, Boiten J, van Oostenbrugge RJ, Al-Shahi Salman R, Lavados PM, Rinkel GJ, van den Bergh WM, MASH-2 Study Group (2012). Magnesium for aneurysmal subarachnoid haemorrhage (MASH-2): a randomised placebo-controlled trial. Lancet.

[13] Veyna RS, Seyfried D, Burke DG, Zimmerman C, Mlynarek M, Nichols V, Marrocco A, Thomas AJ, Mitsias PD, Malik GM (2002). Magnesium sulfate therapy after aneurysmal subarachnoid hemorrhage. J Neurosurg.

[14] van den Bergh WM, Algra A, van Kooten F, Dirven CM, van Gijn J, Vermeulen M, Rinkel GJ, MASH Study Group (2005). Magnesium sulfate in aneurysmal subarachnoid hemorrhage: a randomized controlled trial. Stroke.

[15] Wong GK, Poon WS, Chan MT, Boet R, Gin T, Ng SC, Zee BC, IMASH Investigators (2010). Intravenous magnesium sulphate for aneurysmal subarachnoid hemorrhage (IMASH): a randomized, double-blinded, placebo-controlled, multicenter phase III trial. Stroke.

[16] Wong GK, Chan MT, Boet R, Poon WS, Gin T (2006). Intravenous magnesium sulfate after aneurysmal subarachnoid hemorrhage: a prospective randomized pilot study. J Neurosurg Anesthesiol.

[17] Muroi C, Terzic A, Fortunati M, Yonekawa Y, Keller E (2008). Magnesium sulfate in the management of patients with aneurysmal subarachnoid hemorrhage: a randomized, placebo-controlled, dose-adapted trial. Surg Neurol.

[18] Westermaier T, Stetter C, Vince GH, Pham M, Tejon JP, Eriskat J, Kunze E, Matthies C, Ernestus RI, Solymosi L, Roosen K (2010). Prophylactic intravenous magnesium sulfate for treatment of aneurysmal subarachnoid hemorrhage: a randomized, placebo-controlled, clinical study. Crit Care Med.

[19] Chang JJ, Mack WJ, Saver JL, Sanossian N (2014). Magnesium: potential roles in neurovascular disease. Front Neurol.

[20] van Norden AG, van den Bergh WM, Rinkel GJ (2005). Dose evaluation for long-term magnesium treatment in aneurysmal subarachnoid haemorrhage. J Clin Pharm Ther.

[21] Sehba FA, Hou J, Pluta RM, Zhang JH (2012). The importance of early brain injury after subarachnoid hemorrhage. Prog Neurobiol.

[22] Sehba FA, Pluta RM, Zhang JH (2011). Metamorphosis of subarachnoid hemorrhage research: from delayed vasospasm to early brain injury. Mol Neurobiol.

[23] Mori K, Yamamoto T, Nakao Y, Osada H, Hara Y, Oyama K, Esaki T (2009). Initial clinical experience of vasodilatory effect of intra-cisternal infusion of magnesium sulfate for the treatment of cerebral vasospasm after aneurysmal subarachnoid hemorrhage. Neurol Med Chir (Tokyo).

[24] Mori K, Yamamoto T, Miyazaki M, Hara Y, Aiko Y, Koike N, Sakamoto S, Nakao Y, Esaki T (2011). Optimal cerebrospinal fluid magnesium ion concentration for vasodilatory effect and duration after intracisternal injection of magnesium sulfate solution in a canine subarachnoid hemorrhage model. J Neurosurg.

[25] Takeuchi S, Wada K, Nagatani K, Otani N, Mori K (2012). Magnesium for aneurysmal subarachnoid haemorrhage. Lancet.

[26] Ayer RE, Zhang JH (2008). Oxidative stress in subarachnoid haemorrhage: significance in acute brain injury and vasospasm. Acta Neurochir Suppl.

[27] Yuksel S, Tosun YB, Cahill J, Solaroglu I (2012). Early brain injury following aneurysmal subarachnoid hemorrhage: emphasis on cellular apoptosis. Turk Neurosurg.

[28] Caner B, Hou J, Altay O, Fujii M, Zhang JH (2012). Transition of research focus from vasospasm to early brain injury after subarachnoid hemorrhage. J Neurochem.

[29] Ostrowski RP, Tang J, Zhang JH (2006). Hyperbaric oxygen suppresses NADPH oxidase in a rat subarachnoid hemorrhage model. Stroke.

[30] Dixon BJ, Tang J, Zhang JH (2013). The evolution of molecular hydrogen: a noteworthy potential therapy with clinical significance. Med Gas Res.

[31] Ohsawa I, Ishikawa M, Takahashi K, Watanabe M, Nishimaki K, Yamagata K, Katsura K, Katayama Y, Asoh S, Ohta S (2007). Hydrogen acts as a therapeutic antioxidant by selectively reducing cytotoxic oxygen radicals. Nat Med.

[32] Takeuchi S, Wada K, Nagatani K, Osada H, Otani N, Nawashiro H (2012). Hydrogen may inhibit collagen-induced platelet aggregation: an ex vivo and in vivo study. Intern Med.

[33] Nagatani K, Nawashiro H, Takeuchi S, Tomura S, Otani N, Osada H, Wada K, Katoh H, Tsuzuki N, Mori K (2013). Safety of intravenous administration of hydrogen-enriched fluid in patients with acute cerebral ischemia: initial clinical studies. Med Gas Res.

[34] Nagatani K, Wada K, Takeuchi S, Kobayashi H, Uozumi Y, Otani N, Fujita M, Tachibana S, Nawashiro H (2012). Effect of hydrogen gas on the survival rate of mice following global cerebral ischemia. Shock.

[35] Ishibashi T, Sato B, Rikitake M, Seo T, Kurokawa R, Hara Y, Naritomi Y, Hara H, Nagao T (2012). Consumption of water containing a high concentration of molecular hydrogen reduces oxidative stress and disease activity in patients with rheumatoid arthritis: an open-label pilot study. Med Gas Res.

[36] Sun Q, Kawamura T, Masutani K, Peng X, Sun Q, Stolz DB, Pribis JP, Billiar TR, Sun X, Bermudez CA, Toyoda Y, Nakao A (2012). Oral intake of hydrogen-rich water inhibits intimal hyperplasia in arterialized vein grafts in rats. Cardiovasc Res.

[37] Chen CH, Manaenko A, Zhan Y, Liu WW, Ostrowki RP, Tang J, Zhang JH (2010). Hydrogen gas reduced acute hyperglycemia-enhanced hemorrhagic transformation in a focal ischemia rat model. Neuroscience.

[38] Huang CS, Kawamura T, Toyoda Y, Nakao A (2010). Recent advances in hydrogen research as a therapeutic medical gas. Free Radic Res.

[39] Zhuang Z, Zhou ML, You WC, Zhu L, Ma CY, Sun XJ, Shi JX (2012). Hydrogen-rich saline alleviates early brain injury via reducing oxidative stress and brain edema following experimental subarachnoid hemorrhage in rabbits. BMC Neurosci.

[40] Hong Y, Guo S, Chen S, Sun C, Zhang J, Sun X (2012). Beneficial effect of hydrogen-rich saline on cerebral vasospasm after experimental subarachnoid hemorrhage in rats. J Neurosci Res.

[41] Zhuang Z, Sun XJ, Zhang X, Liu HD, You WC, Ma CY, Zhu L, Zhou ML, Shi JX (2013). Nuclear factor-κB/Bcl-XL pathway is involved in the protective effect of hydrogen-rich saline on the brain following experimental subarachnoid hemorrhage in rabbits. J Neurosci Res.

[42] Hong Y, Shao A, Wang J, Chen S, Wu H, McBride DW, Wu Q, Sun X, Zhang J (2014). Neuroprotective effect of hydrogen-rich saline against neurologic damage and apoptosis in early brain injury following subarachnoid hemorrhage: possible role of the Akt/GSK3β signaling pathway. PLoS One.

[43] Connolly ES, Rabinstein AA, Carhuapoma JR, Derdeyn CP, Dion J, Higashida RT, Hoh BL, Kirkness CJ, Naidech AM, Ogilvy CS, Patel AB, Thompson BG, Vespa P, American Heart Association Stroke Council; Council on Cardiovascular Radiology and Intervention; Council on Cardiovascular Nursing; Council on Cardiovascular Surgery and Anesthesia; Council on Clinical Cardiology (2012). Guidelines for the management of aneurysmal subarachnoid hemorrhage: a guideline for healthcare professionals from the American Heart Association/American Stroke Association. Stroke.

[44] Nakamura T, Matsui T, Hosono A, Okano A, Fujisawa N, Tsuchiya T, Indo M, Suzuki Y, Oya S, Chang HS (2013). Beneficial effect of selective intra-arterial infusion of fasudil hydrochloride as a treatment of symptomatic vasospasm following SAH. Acta Neurochir Suppl.

[45] Tachibana E, Harada T, Shibuya M, Saito K, Takayasu M, Suzuki Y, Yoshida J (1999). Intra-arterial infusion of fasudil hydrochloride for treating vasospasm following subarachnoid haemorrhage. Acta Neurochir (Wien).

[46] Kaneda K, Fujita M, Yamashita S, Kaneko T, Kawamura Y, Izumi T, Tsuruta R, Kasaoka S, Maekawa T (2010). Prognostic value of biochemical markers of brain damage and oxidative stress in post-surgical aneurysmal subarachnoid hemorrhage patients. Brain Res Bull.

[47] Moritz S, Warnat J, Bele S, Graf BM, Woertgen C (2010). The prognostic value of NSE and S100B from serum and cerebrospinal fluid in patients with spontaneous subarachnoid hemorrhage. J Neurosurg Anesthesiol.

[48] Fountas KN, Tasiou A, Kapsalaki EZ, Paterakis KN, Grigorian AA, Lee GP, Robinson JS (2009). Serum and cerebrospinal fluid C-reactive protein levels as predictors of vasospasm in aneurysmal subarachnoid hemorrhage. Clinical article. Neurosurg Focus.

[49] Frontera JA, Fernandez A, Schmidt JM, Claassen J, Wartenberg KE, Badjatia N, Connolly ES, Mayer SA (2009). Defining vasospasm after subarachnoid hemorrhage: what is the most clinically relevant definition?. Stroke.

[50] Mori K, Yamamoto T, Miyazaki M, Hara Y, Koike N, Nakao Y (2013). Potential risk of artificial cerebrospinal fluid solution without magnesium ion for cerebral irrigation and perfusion in neurosurgical practice. Neurol Med Chir (Tokyo).

